# Decreased Expression of Programmed Death Ligand-L1 by Seven in Absentia Homolog 2 in Cholangiocarcinoma Enhances T-Cell–Mediated Antitumor Activity

**DOI:** 10.3389/fimmu.2022.845193

**Published:** 2022-01-27

**Authors:** Hao Zheng, Wen-juan Zheng, Zhen-guang Wang, Yuan-ping Tao, Zhi-ping Huang, Le Yang, Liu Ouyang, Zhi-qing Duan, Yi-nuo Zhang, Bo-ning Chen, Dai-min Xiang, Gang Jin, Lu Fang, Fan Zhou, Bo Liang

**Affiliations:** ^1^ Department of General Surgery, The Second Affiliated Hospital of Nanchang University, Nanchang, China; ^2^ Department of Reproductive Heredity Center, Changhai Hospital, Second Military Medical University, Shanghai, China; ^3^ Third Department of Hepatic Surgery, Eastern Hepatobiliary Surgery Hospital, Second Military Medical University, Shanghai, China; ^4^ Key Laboratory of Signaling Regulation and Targeting Therapy of Liver Cancer (SMMU), Ministry of Education, Shanghai, China; ^5^ Shanghai Key Laboratory of Hepatobiliary Tumor Biology (EHBH), Shanghai, China; ^6^ National Liver Tissue Bank, Eastern Hepatobiliary Surgery Hospital, Second Military Medical University, Shanghai, China; ^7^ Department of Hepatobiliary Surgery, General Hospital of Southern Theatre Command, Guangzhou, China; ^8^ Department of Hepatobiliary Pancreatic Surgery, Changhai Hospital of Second Military Medical University, Shanghai, China; ^9^ State Key Laboratory of Oncogenes and Related Genes, Shanghai Cancer Institute, Renji Hospital, Shanghai Jiao Tong University School of Medicine, Shanghai, China

**Keywords:** N6-methyladenosine, METTL14, Siah2, PD-L1, immunotherapy

## Abstract

N6-methyladenosine (m6A) has been reported as an important mechanism of post-transcriptional regulation. Programmed death ligand 1 (PD-L1) is a primary immune inhibitory molecule expressed on tumor cells that promotes immune evasion. In addition, seven in absentia homolog 2 (Siah2), a RING E3 ubiquitin ligase, has been involved in tumorigenesis and cancer progression. However, the role of m6A-METTL14-Siah2-PD-L1 axis in immunotherapy remains to be elucidated. In this study, we showed that METTL14, a component of the m^6^A methyltransferase complex, induced Siah2 expression in cholangiocarcinoma (CCA). METTL14 was shown to enrich m^6^A modifications in the 3’UTR region of the Siah2 mRNA, thereby promoting its degradation in an YTHDF2-dependent manner. Furthermore, co-immunoprecipitation experiments demonstrated that Siah2 interacted with PD-L1 by promoting its K63-linked ubiquitination. We also observed that *in vitro* and *in vivo* Siah2 knockdown inhibited T cells expansion and cytotoxicity by sustaining tumor cell PD-L1 expression. The METTL14-Siah2-PD-L1–regulating axis was further confirmed in human CCA specimens. Analysis of specimens from patients receiving anti-PD1 immunotherapy suggested that tumors with low Siah2 levels were more sensitive to anti-PD1 immunotherapy. Taken together, our results evidenced a new regulatory mechanism of Siah2 by METTL14-induced mRNA epigenetic modification and the potential role of Siah2 in cancer immunotherapy.

## Introduction

Cholangiocarcinoma (CCA) is the second most common primary malignant tumor of liver cancers, whose recurrence rate is significantly higher than that of hepatocellular carcinoma. The overall median survival time of ICC patients is less than one year (1–3). For patients with advanced or inoperable CCA, gemcitabine–cisplatin combination is recommended as systemic therapy but with marginal improvement in overall survival. Unfortunately, we currently lack of a no standard second-line therapy (4). Thus, it is urgent to develop new strategies to prevent tumor progression and improve the prognosis of CCA patients.

RNA N6-methyladenosine (m6A) is the most abundant internal modification in mRNAs, which is widely present in mammal cells and mainly present in the last exon of mRNA (5). Its methylation is regulated by “writer,” “eraser,” and “reader” proteins (6). In addition, m6A methyltransferase complex is mainly composed of METTL3 (methyltransferase-like 3), METTL14 (methyltransferase-like 14), and WTAP (Wilms tumor 1-associated protein), which act as “writer” to catalyze methylation processes, whereas FTO (fat mass and obesity associated protein) and ALKBH5 (alkB homolog 5) act as demethylases (“eraser”). The ultimate fate of m6A methylated mRNA depends on the “reader” (e.g., YTHDF1, YTHDF2, and YTHDF3) that recognizes them, affecting mRNA translation, stability, splicing, and nuclear transportation (7). Numerous m6A targets have been involved in cell tissue development and stem cell self-renewal and differentiation, circadian rhythm regulation, T-cell homeostasis, mouse fertility, postnatal development of the mouse cerebellum, innate immune response, ultraviolet-induced DNA damage response, and dendritic cell antigen presentation (8–10). However, the possible role of m6A modification in tumor-immune microenvironment and in CCA remains to be elucidated.

Programmed death-ligand 1 (PD-L1, also named B7-H1) is a major inhibitory immune checkpoint molecule that facilitates tumor evasion of immune surveillance by binding to programmed death receptor-1 (PD-1) on T cells, which induces T lymphocytes apoptosis, anergy, and functional exhaustion (11). The use of anti-PD-1/PD-L1 immunotherapy to block such an interaction, activating T-cell immunity has produced significant results in various types of cancer (12). In this regard, the anti-PD-1 antibody pembrolizumab has been approved by the FDA for the treatment of patients with unresectable or metastatic mismatch repair deficient and/or microsatellite-high solid tumors, including CCA, which progressed after prior therapy (13). The objective response rate of pembrolizumab is still limited but a better outcome was associated with high PD-L1 expression in CCA (14). Understanding the regulation mechanism underlying PD-L1 expression is essential for developing efficient combination immunotherapeutic strategies (15). It has been reported that PD-L1 expression is regulated by genetic alterations (e.g., rearrangements in the 30 UTR PD-L1 mRNA), tumor-intrinsic oncogenic pathways (e.g., RAS-MEK-ERK, CDK5, PI3K-AKT, HIF-1a), and posttranslational modifications (e.g., CMTM6/CMTM4, HIP1R, CUL3), whereas the exact regulation mechanism of PD-L1 on protein level is still unknown (16).

SIAH1 and Siah2 are highly homologous RING domain E3 ligases that often target common proteins for degradation (17). Two SIAH homologs in humans (SIAH1 and Siah2) and three homologs in mice (Siah1a, Siah1b, and Siah2) have been reported (18). SIAH proteins contain an N-terminal RING domain and a C-terminal substrate-binding domain (SBD) (19), which are involved in ubiquitination of diverse substrates and other biological processes such as RAS signaling, DNA damage, hypoxia, p38/JNK/NF-кB pathways, and transcription (20–22). In particular, Siah2 plays a critical role in tumorigenesis and cancer progression (23). Numerous studies have determined that Siah2 exerts its biological function, depending on the tumor development stage (23). However, the exact regulation mechanism of Siah2-regulated PD-L1 on protein level in CCA is still unclear.

In the present study, we showed an epigenetic regulating mechanism of Siah2 on mRNA level by METTL14, which depended on m6A modification. We further unveiled a complex role of Siah2 in immune microenvironment and demonstrated that tumors with low Siah2 expression pattern were more sensitive to anti-PD-1 immunotherapy. Taken together, our results evidenced a new regulation mechanism of PD-L1 and unveiled a role of METTL14-Siah2 axis in immunotherapy, which might contribute to CCA immunotherapy.

## Materials and Methods

### Patients and Tissue Specimens

Tissue specimens were obtained from the Eastern Hepatobiliary Surgery Hospital (Shanghai, China) and the Changhai Hospital (Shanghai, China). Written informed consent was obtained from patients. The procedure of human specimen collection was approved by the Ethics Committees of these Hospitals.

### Lentiviral Transduction for Stable Cell Line

Lentiviruses used in this study were purchased from Genechem (Shanghai, China). Lentiviral infection was performed according to manufacturers’ instructions. In brief, RBE and HUCCT1 cells were seeded in 6-well plates in 2 mL complete medium and transduced by lentivirus at a multiplicity of infection of 15 to 20 in the presence of the HitransG A reagent (Genechem). We then used 5 g/mL puromycin (Selleck, Shanghai, China) for the selection of transduced cells. After continuous selection for approximately two weeks, the surviving cell colonies were expanded. The efficacy of overexpression and knockdown was verified by Western blotting.

### shRNA Transfection

shRNAs were synthesized by Biotend Biotechnology (Shanghai, China). For siRNA transfection, RBE and HUCCT1 cells were cultured in 12- or 6- well plates to 60%-70% confluency. One hundred nanomoles of siRNA or siRNA negative control (NC) were transfected to cells using Lipofectamine 2000 (ThermoFisher Scientific, Waltham, MA, USA), following manufacturer’s instructions. After 48 h or 72 h of transfection, cells were used for subsequent experiments. Sequences of shRNA seed sequence were provided in **Table S1**.

### Western Blot

Western blotting analysis was performed as previously reported (24). In brief, cells were lysed in RIPA lysis buffer (Beyotime Biotechnology, Shanghai, China) with 1 mM PMSF on ice for 10 min. Protein concentrations were measured by the Pierce™ BCA Protein Assay Kit (ThermoFisher Scientific). Equal amounts of protein were separated by SDS-PAGE and transferred onto 0.22 μm nitrocellulose membranes (Millipore, Cork, Ireland). Membranes were then incubated with anti-primary antibodies (LI-COR Biosciences, Lincoln, NE, USA) overnight at 4°C, followed by incubation with IRDye 800 goat anti-rabbit antibodies (LI-COR Biosciences) for 1 h at room temperature. After removing the unbound antibodies, the labelled bands were scanned in the Odyssey^®^ CLx Infrared Imaging System (LI-COR Biosciences). Anti-primary antibodies are listed in **Table S2**.

### RNA Isolation and Quantitative Real Time PCR (RT-PCR)

TRIzol™ Reagent (Invitrogen, Carlsbad, CA, USA) was used to isolate total RNA from samples. Quantitative RT-PCR was performed as previously reported (25) on an ABI 7300 Fast Real-Time PCR System (Applied Biosystems, Foster City, CA), using the SYBR Green PCR kit (Applied TaKaRa, Otsu, Shiga, Japan). The expression levels of the target genes were normalized against GAPDH and analyzed by the Delta-Delta-Ct (△△Ct) method (3). Primers for quantitative RT-PCR are listed in **Table S3**.

### Nuclear and Cytoplasmic Fractions Assay

Nuclear and cytoplasmic fractions were extracted by the PARISTM Kit (Ambion, Austin, TX, USA), following manufacturer’s instructions and performed as previously reported (25). GAPDH and histone H3 (Bioss Antibodies Inc., Woburn, MA, USA) protein levels detection by Western blotting was used to confirm the successful cellular fraction.

### Cell Proliferation Assay

Relevant cells were seeded in 96-well plates at a density of 1×10^3^ cells/well. Cell viability was determined by the CCK-8 assay kit, following manufacturer’s instructions at indicated timepoints after seeding. Results were analyzed using the GraphPad Prism 8.0 software (GraphPad Software Inc., San Diego, CA, USA).

### Quantitative Analysis of Immunohistochemistry (IHC) Staining

IHC was regularly performed and H-Score was calculated for quantitative analysis of IHC staining, as previously reported (26). The photographs of the stained sections were obtained by the Leica Aperio AT Turbo Digital Slide Scanning System (Leica Biosystems, MA, USA).

### hPBMC+ Humanized mouse models

hPBMC+ humanized NCG mice were purchased from the Model Animal Research Center of Nanjing University and were constructed as previously reported (25). Immune cell percentages were detected by flow cytometry 3 wk after hPBMC+ cell injection, hPBMC+ humanized NCG mice were randomly assigned into experiment groups. Indicated CCA cells with LV-NC/LV-SIAH2 of 5×106 were injected into the right flank of hPBMC+ humanized NCG mice. Tumor volume was calculated by the following formula: volume = ab2/2 (a, the longer axis; b, the shorter axis). After 35 d of cell inoculation, hPBMC+ humanized NCG mice were euthanized and tumor-infiltrating leukocytes were isolated and subjected to CyTOF analysis. The animal study was conducted in conformity with NIH and the Second Affiliated Hospital of Nanchang University, Servicebio Animal Welfare guidelines and approved by Wuhan Servicebio Technology Co., Ltd., China.

### PD-L1 Detection on Cell Surface

PCCA#1/#2 Cells were digested and collected. After washing with staining buffer, 1×10^6^ cells were suspended in 100 mL of staining buffer and incubated at 4°C for 30 min with PE Mouse Anti-Human PD-L1 (MIH1; BD Biosciences, San Jose, CA, USA) or APC Mouse Anti-Human PD-L1 (MIH1; BD Biosciences). After washing with staining buffer, samples were subjected to flow cytometry analysis with a BD LSRFortessa cell analyzer (BD Biosciences). Data were then analyzed with the FlowJo vX.07 software (Tree Star, San Carlos, CA, USA).

### RNA Immunoprecipitation

RNA immunoprecipitation assay was conducted with the Magna RIP RNA-Binding Protein Immunoprecipitation Kit (Millipore), following manufacturer’s instructions and performed as previously reported (25). RNA quality was evaluated on a Nanodrop2000 (Thermo Scientific, Wilmington, DE, USA).

### mRNA Stability Detection

mRNA stability assays were performed as previously reported (25). Cells were cultured overnight, after which they were treated with 5 mg/mL actinomycin D at indicated times. Total RNA was extracted by TRIzol (Invitrogen). Quantitative RT-PCR was performed to determine the relative level of indicated mRNA.

### Isolation and Activation of Human Peripheral Blood Mononuclear Cells (HPBMC)

HPBMC from health donors were provided by the Changhai Hospital (Shanghai, China) and isolated with Lymphoprep (Stemcell Technologies, Vancouver, Canada), following manufacturer’s instructions and performed as previously reported (23).

### Statistical Analysis

Statistical tests were two sided and values were expressed as mean ± SD, unless otherwise specified. The analysis was performed using the GraphPad Prism8 software (GraphPad Software Inc.). Unpaired Student *t* test or paired Student *t* test was used to compare between two experimental groups. Cumulative survival time was estimated by the Kaplan–Meier method, and the log-rank test was applied to compare groups. P ≤ 0.05 was considered statistically significant. We included all animal data.

Details of other regular experiments were provided in **Supplementary Materials and Methods**.

## Results

### Siah2 Is a Direct Target of METTL14-Induced m6A Modification in CCA

**Figure 1:**
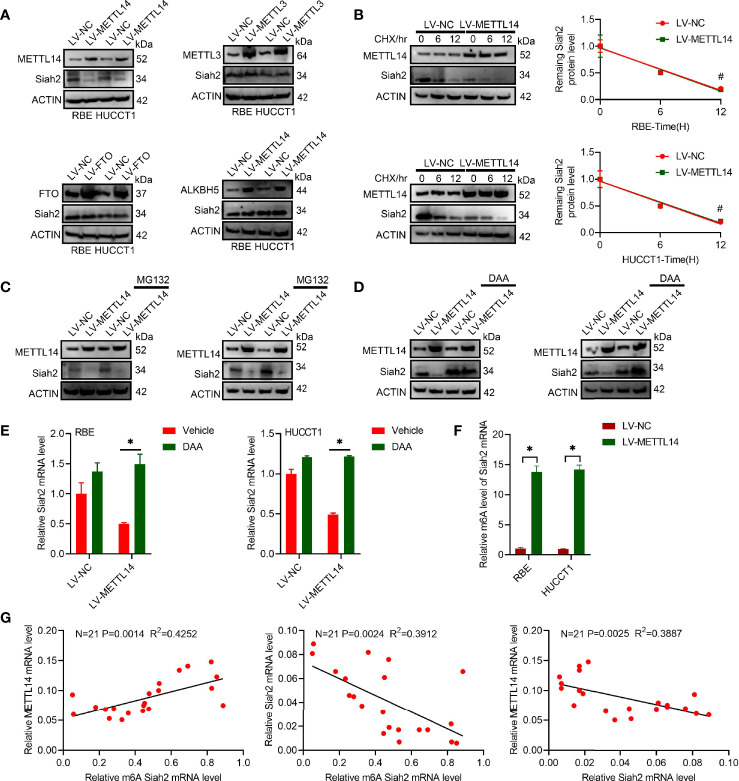
Siah2 is a direct target of METTL14-induced m6A modification in CCA. **(A)**, Western blot analysis for Siah2 expression in CCA cell lines with stable METTL14, METTL3, ALKBH5, or overexpressed FTO. **(B)**, The rate of Siah2 protein degradation was checked by Western blotting in CCA cells with METTL14 knocked down upon CHX (protein synthesis inhibitor) treatment. **(C)**. Western blot analysis for Siah2 expression in CCA cells with METTL14 knocked down upon MG132 treatment. **(D, E)**, Western blot and RT-PCR analysis for Siah2 expression in CCA cells with LV-NC/-METTL14 upon DDA 0–25 mmol/L treatment for 24 h. **(F)**, m6A fold enrichment of Siah2 total mRNA detected by MeRIP quantitative RT-PCR of fragmented mRNA with indicated primers. **(G)**, Correlation between METTL14 MRNA level, M6A level of Siah2, and Siah2 MRNA level in CCA tissues. Mean ± SEM of three independent experiments. *P < 0.05; ^#^P > 0.05.

We found that increased METTL14 caused a significant reduction on Siah2 protein levels in human CCA cell lines, whereas increased FTO, METTL3, or ALKBH5 did not affect its levels (**Figure 1A**). This indicated that Siah2 was specifically regulated by METTL14 but not by FTO, METTL3, or ALKBH5 in CCA. The fact that LV-METTL14 did not affect Siah2 protein degradation rate was demonstrated by the use of cycloheximide (CHX) and MG132 treatments (**Figures 1B, C**). Furthermore, we reversed LV-METTL14-restrained Siah2 protein and mRNA level, using the global methylation inhibitor 3-deazaadenosine (DAA) (**Figures 1D, E**), thus indicating that METTL14 controls Siah2 expression depending on its demethylation activity. The m6A level of Siah2 total mRNA was significantly increased in LV-METTL14 cells (**Figure 1F**).

The Siah2 m6A level was positively associated with METTL14 mRNA and negatively associate with Siah2 mRNA. METTL14 mRNA was negatively associated with Siah2 mRNA in 21 CCA tissues (**Figure 1G**). Taken together, these data indicated that METTL14 regulated the level of m6A modification in CCA.

### METTL14 Promotes Siah2 mRNA Degradation in CCA

**Figure 2:**
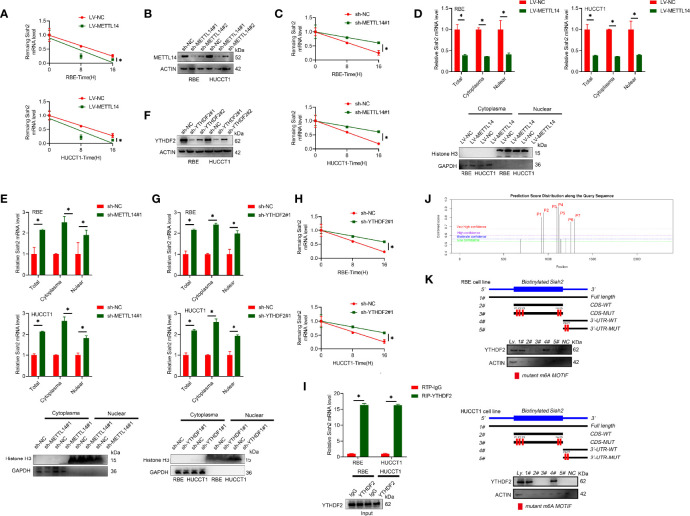
METTL14 promotes Siah2 mRNA degradation in CCA. **(A)**, The stability of Siah2 mRNA in CCA cells with LV-NV or LV-METTL14 was detected by quantitative RT-PCR upon actinomycin D 10 mg/ml treatment. **(B)**, Western blot analysis for METTL14 expression in CCA cell lines with stable METTL14 knockdown. **(C)**, The stability of Siah2 mRNA in CCA cells with sh-NC or sh-METTL14 was detected by quantitative RT-PCR upon 10 mg/mL actinomycin D treatment. **(D, E)**, Quantification of Siah2 mRNA level in total, nuclear, and cytoplasmic fractions by quantitative RT-PCR in CCA cells. Nuclear and cytoplasmic fractions were evaluated by Western blot analysis. **(F)**, The successful knockdown of YTHDF2 in CCA cells was confirmed by Western blot analysis. **(G)**, The expression of Siah2 mRNA was detected by quantitative RT-PCR in CCA cells transfected with sh-NC or sh-YTHDF2. **(H)**,The stability of Siah2 mRNA was detected by quantitative RT-PCR in CCA cells transfected with sh-NC or sh-YTHDF2 upon 10 mg/mL actinomycin D treatment. **(I)**,RNA immunoprecipitation assay detected the interaction between YTHDF2 and RNA in CCA cells. RNA enrichment was measured by quantitative RT-PCR. RIP with non-specific IgG was set as control. Western blot of YTHDF2 showed equal amount of input YTHDF2 protein in two groups. **(J)**, Bioinformatics predicted that Siah2 may produce m6A methylation at multiple sites. **(K)**, Immunoblotting of YTHDF2 with cell lysate(Ly.),full-length biotinylated-Siah2(#1),the Siah2 CDS region with or without m6A motif mutation(#2,#3),the Siah2 3′-UTR region with or without m6A motif mutation(#4,#5),and beads only(NC)in CCA cells Mean ± SEM of three independent experiments. *P < 0.05.

It has been reported that m6A modification affects mRNA stability of targeted mRNAs, depending on the distinct proteins (YTHDF1, YTHDF2, and YTHDF3) that recognize them. Blocking new RNA synthesis with actinomycin D indicated that METTL14-stable overexpression significantly increased Siah2 mRNA stability, whereas METTL14 knockdown inhibited Siah2 mRNA degradation (**Figures 2A–C**). Furthermore, METTL14 deficiency significantly increased and METTL14 overexpression significantly decreased the level of Siah2 mRNA in cytoplasmic and nuclear fractions (**Figures 2D, E**). YTHDF2 has been reported to specifically recognize and bind m6A-containing RNAs, regulating mRNA stability (25). As shown in **Figures 2F–H**, YTHDF2 knockdown significantly increased Siah2 mRNA level and stability of PD-L1 mRNA. Following, the interaction between YTHDF2 and Siah2 mRNA was confirmed by the RIP assay (**Figure 2I**). Based on SRAMP (A sequence-based N6-methyladenosine (m6A) modification site predictor) database, seven peaks were identified in Siah2 mRNA, with peaks 6 to 7 on the 3’ UTR region and peaks 1 to 5 on the coding sequence region (**Figure 2J**). Notably, the RNA pull-down assays verified that YTHDF2 predominantly bound to the Siah2 3′-UTR region, instead of CDS in CCA cells, and the specific binding was significantly impaired after m6A peak depletion(**Figure 2K**). These data suggested that METTL14 regulated the stability of Siah2 mRNA in an YTHDF2-dependent manner.

### Siah2 Regulates PD-L1 Stability in CCA

**Figure 3:**
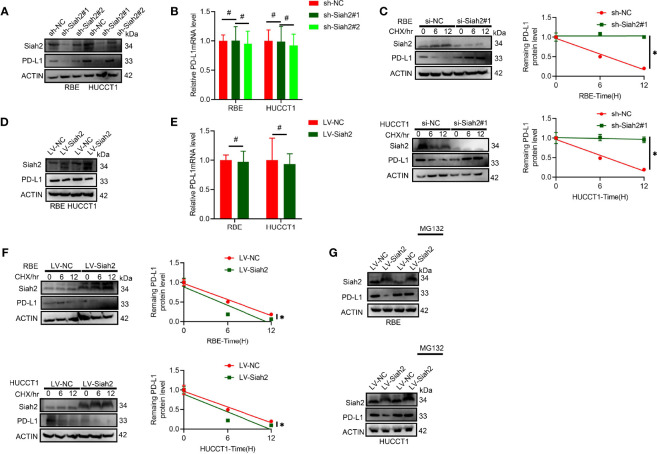
Siah2 regulates PD-L1 stability in CCA. **(A, B)**, Depletion of Siah2 increases the protein level but not the mRNA level, of PD-L1 in CCA cells. **(C)**, Depletion of Siah2 increases the protein stability of PD-L1 under CHX treatment. **(D, E)**, Overexpression of Siah2 decreases the protein level, but not the mRNA level, of PD-L1 in CCA cells. **(F)**, Overexpression of Siah2 decreases the protein stability of PD-L1 under CHX treatment. **(G)**, The protein level of Siah2 was evaluated by Western blot in CCA cells with LV-NC or LV-METTL14 upon MG132 treatment. Mean ± SEM of three independent experiments. *P < 0.05; ^#^P > 0.05.

It was found that protein level but not the mRNA level of PD-L1 increased in CCA cells with Siah2 knocked down (**Figures 3A, B**). We further tested whether inhibition of SIAH2 affected PD-L1 protein stability by blocking *de novo* protein synthesis with CHX. As seen in **Figure 3C**, depletion of Siah2 increased the protein stability of PD-L1 in CCA cells. In contrast, increased Siah2 decreased PD-L1 protein level and PDL1 protein stability, whereas mRNA level was not altered in the Siah2 increased group (**Figures 3D–F**). Moreover, the proteasome inhibitor MG132 rescued the effect of Siah2 on PD-L1, suggesting that Siah2 promotes proteasomal degradation of PD-L1 (**Figure 3G**). These results strongly suggest that Siah2 promotes PD-L1 proteasomal degradation.

### Siah2 Physically Interacts With PD-L1 and Augments the K63-Linked Ubiquitination of PD-L1 in CCA

**Figure 4:**
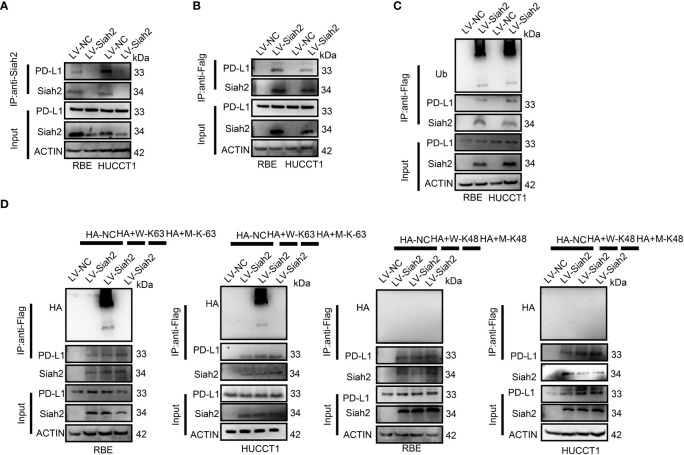
Siah2 physically interacts with PD-L1 and increases the K63-linked ubiquitination of PD-L1 in CCA. **(A)**, CCA cells were transfected with HA-Siah2 and Flag-PD-L1 for 36 h. Immunoprecipitation was performed with anti-Siah2 and analyzed by immunoblotting. **(B)**, CCA cells were transfected with HA-Siah2 and Flag-PD-L1 for 36 h. Immunoprecipitation was performed with anti-HA and analyzed by immunoblotting. **(C)**, CCA cells with LV-NC/LV-Siah2 were treated with MG132 for 8 h before harvesting, after which cells were harvested in lysis buffer. Supernatants were incubated with the appropriate antibodies overnight. Ubiquitin-modified proteins were analyzed with anti-Ub antibodies. **(D)**, CCA cells with LV-NC/LV-Siah2 were treated with MG132 for 8 h before harvesting, after which cells were harvested in lysis buffer. Supernatants were incubated with the appropriate antibodies overnight. Ubiquitin-modified proteins were analyzed with anti-lysine 48(K48)–linked polyubiquitination or K63-linked polyubiquitination.

We tested whether Siah2 may interact with PD-L1 in CCA. As shown in **Figures 4A, B**, co-immunoprecipitation assays evidenced that endogenous and exogenous Siah2 specifically interact with PD-L1. Thus increased Siah2 in CCA cells enhanced the ubiquitination of PD-L1 in CCA (**Figure 4C**). We also observed that Siah2 promoted the K63-linked ubiquitination of PD-L1 but not K48-linked ubiquitination (**Figure 4D**). These data suggest that Siah2 physically interacts with PD-L1 and increases the K63-linked ubiquitination of PD-L1 in CCA.

### *In Vitro* Siah2 Enhanced Antitumor T-Cell Immunity

**Figure 5:**
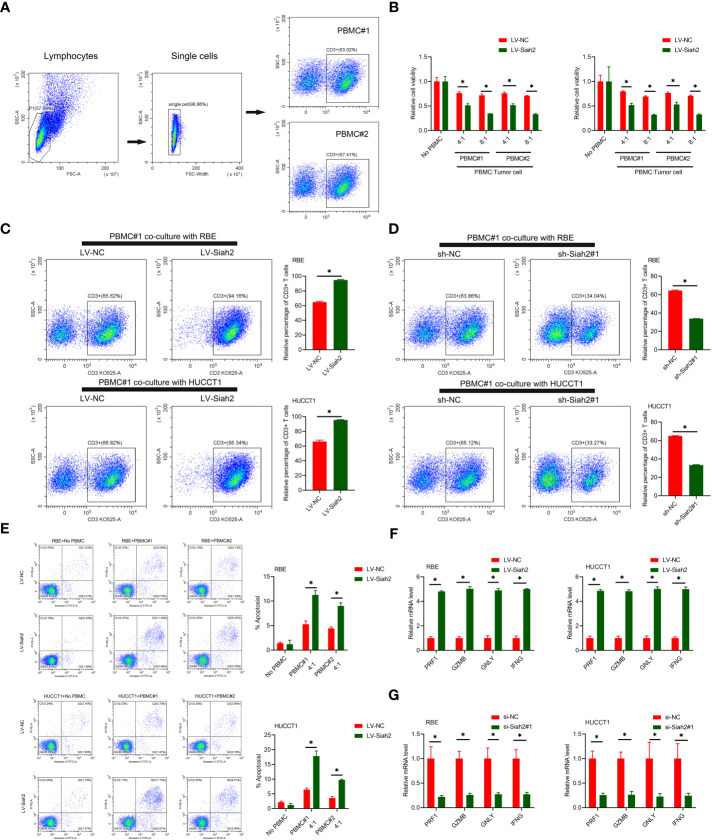
Siah2 enhanced *in vitro* antitumor T-cell activity. **(A)**, Gating strategy and T cell percentage of activated PBMC. **(B)**, CCK8 assay detected the killing of tumor cells by activated HPBMC. CCA cells were co-cultured in the presence or absence of HPBMC for 24 h. Data were normalized to their respective no HPBMC controls. **(C, D)**, Activated HPBMC (#1, #2) were co-cultured with LV-NC/LV-Siah2 **(C)** or sh-NC/sh-Siah2 **(D)** for three days at the ratio of HPBMC to tumor cell number of 4 to 1. The percentage of CD3 in HPBMC was determined by FCM. Representative plots of CD3^+^ T cells are shown. Data were normalized to the control group. **(E)**, Activated HPBMC (#1, #2) were co-cultured with CCA with LV-NC/LV-Siah2 cells for 24 h at the ratio of HPBMC to tumor cell number of 4 to 1. HPBMC were collected and stained with PE-Annexin V and subjected to FCM analysis. The percentage of apoptotic cells was analyzed. **(F, G)**, Quantitative RT-PCR was performed to detect PRF1 (perforin-1), GZMB (granzyme), GNLY (granulysin), and IFNG (IFN-γ) in activated HPBMC co-cultured with CCA cells with LV-NC/LV-Siah2 **(G)** for the indicated time or with si-NC/si-Siah2 cells for 48 h. The ratio of HPBMC to tumor cell number was 4 to 1. Mean ± SEM of three independent experiments. *P < 0.05.

PD-L1 on tumor cells binds to PD-1 on activated T cells, leading to exhaustion and apoptosis of T cells, which has been identified as a key process in tumor cell–mediated immune escape (24). To test whether Siah2 may regulate antitumor T-cell immunity *via* regulating PD-L1, we performed *in vitro* T-cell–mediated killing assays in a co-culture, in which activated HPBMC were cultured in the presence of human CCA cell lines. HPBMC were activated and expanded with anti-CD3/CD28 antibodies and IL-2, before co-culturing with tumor cells. Lymphocytes used in the study consisted of 60% CD3^+^ T cells (**Figure 5A**). CCA cells with LV-Siah2 were more susceptible to T-cell killing (**Figure 5B**). The CD3^+^ cell proportion of HPBMC decreased upon co-culturing with the sh-Siah2 cells group, compared with the sh-NC cells group, and increased upon co-culturing with the LV-Siah2 cell group, compared with the LV-NC cells group (**Figures 5C, D**). Furthermore, the number of apoptotic tumor cells increased in LV-Siah2 cells following HPBMC co-culturing (**Figure 5E**). Moreover, the mRNA levels of PRF1 (perforin 1), GZMB (granzyme B), GNLY (granulysin), and IFN-γ in PBMCs decreased following co-culturing with LV-Siah2 cells, and increased upon co-culturing with shSiah2 cells (**Figures 5F, G**). Taken together, these data suggested that Siah2 deficiency in tumor cells enhanced T-cell–mediated antitumor activity and vice versa.

### *In Vivo* Siah2 Enhanced Antitumor T-Cell Activity

**Figure 6:**
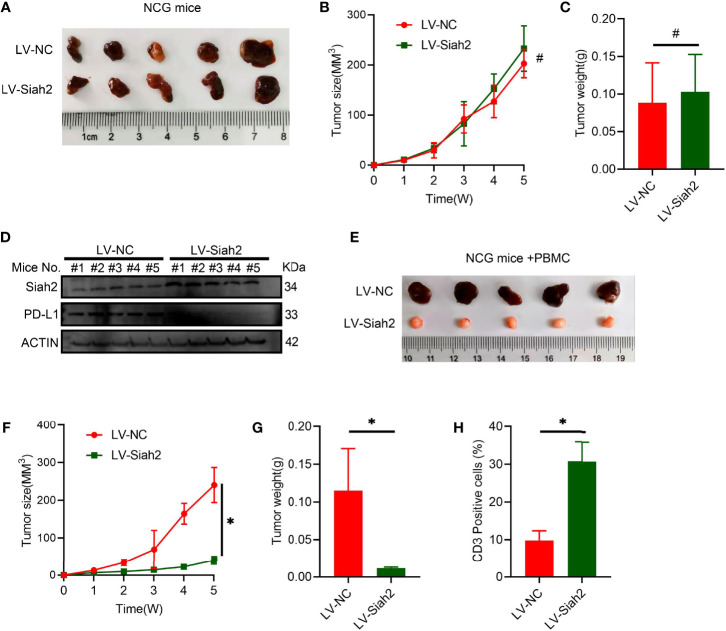
Siah2 enhanced *in vivo* antitumor T-cell activity. **(A–C)**, CCA cells with LV-NC/LV-Siah2 were injected subcutaneously into the right flank of NCG mice to obtain tumor xenografts. Tumor volume **(B)** and tumor weight **(C)** were then measured. n = 5. ns, non-significant. Unpaired *t* test. **(D)**, Western blot analysis detected PD-L1 level in the subcutaneous xenografts described in **(A–C)**. **(E–H)**, CCA cells with LV-NC/LV-Siah2 were injected subcutaneously into the right flank of NCG mice, which were adoptively transferred with activated HPBMC. **(E–G)**, Images of subcutaneous xenografts **(E)**, tumor volume **(F)**, and tumor weight **(G)** are shown. N = 5. **(H)**, FCM detected the percentage of human CD3^+^ T cells in HPBMC of NCG mice. n = 5. Mean ± SEM of three independent experiments. *P < 0.05; ^#^P > 0.05.

To verify the potential *in vivo* effect of Siah2 on CCA progression, we subcutaneously transplanted a xenograft in immunodeficient NCG mice in LV-NC/LV-Siah2 group and did not find a significant difference (**Figures 6A–C**), indicating that Siah2 exerted a marginal *in vivo* effect on CCA cell growth. Moreover, the PD-L1 protein level was downregulated by LV-Siah2 in xenografts (**Figure 6D**). Since we lack of a murine CCA cell line, we further transplanted a subcutaneous xenograft of LV-NC/LV-Siah2 CCA cells in HPBMC-transferred immunodeficient NCG mice. HPBMC were pre-activated and expanded with anit-CD3/CD28 antibodies and IL-2 for 12 d before transferring, and the percentage of CD3^+^ T cells reached 80%. The tumor size in the LV-Siah2 group was significantly smaller than that of the LV-NC group in HPBMC-transferred NCG mice (**Figures 6E–G**). As expected, the percentage of human CD3^+^ cell in HPBMCs was higher in the LV-Siah2 group than that in the LV-NC group (**Figure 6H**).

### Siah2 Enhanced Antitumor T-Cell Activity in a PD-L1–Dependent Manner

**Figure 7:**
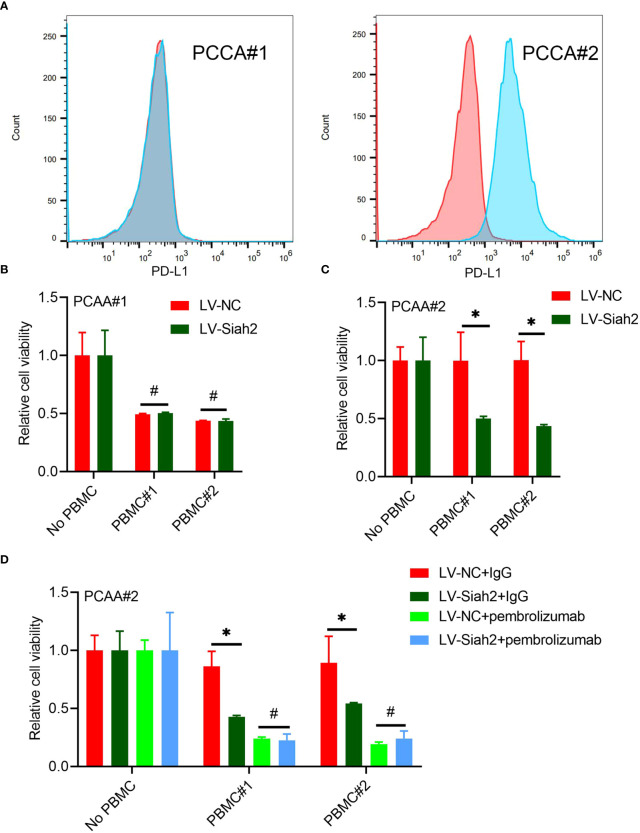
Siah2 enhanced antitumor T-cell immunity in a PD-L1–dependent manner. **(A)**, FCM detected the cell membranous PD-L1 level of PCCA#1 and #2. **(B, C)**, CCK8 assay detects the killing of PCAA#1 LV-NC/LV-Siah2 by activated HPBMC in PCCA#1 and PCCA#2 cells. The ratio of PBMC to tumor cell number was 4 to 1, n = 3. **(D)**, CCK8 assay detected the killing of tumor cells by activated HPBMC after Pembrolizumab or 10 µg/mL IgG treatment. PCAA#2-LV-NC/LV-METTL14 were co-cultured in the presence or absence of HPBMC for 24 h at the ratio of HPBMC to tumor cell number of 4 to 1, n = 3. Mean ± SEM of three independent experiments. *P < 0.05; ^#^P > 0.05.

Flow cytometry detected the membranous distribution of PD-L1 on the primary CCA#1 and CCA#2 cells, indicating a high membranous distribution of PD-L1 on PCCA#2 and absence of PD-L1 membranous distribution on PCCA#1(**Figure 7A**). We did not observe a significant change of tumor cell viability between PCCA#1-shSiah2 and PCCA#1-shCtrl cells in co-culturing with HPBMC (**Figure 7B**). The difference of cell susceptibility to T-cell killing between PCCA#2-LV-NC and PCCA#2-LV-Siah2 was reversed by blocking PD-L1 with pembrolizumab (**Figures 7C, D**), indicating that PD-L1 is required for Siah2-mediated tumor resistance to T-cell antitumor activity.

### The Correlation Between METTL14, Siah2, and PD-L1 in Clinical CCA Specimens

**Figure 8:**
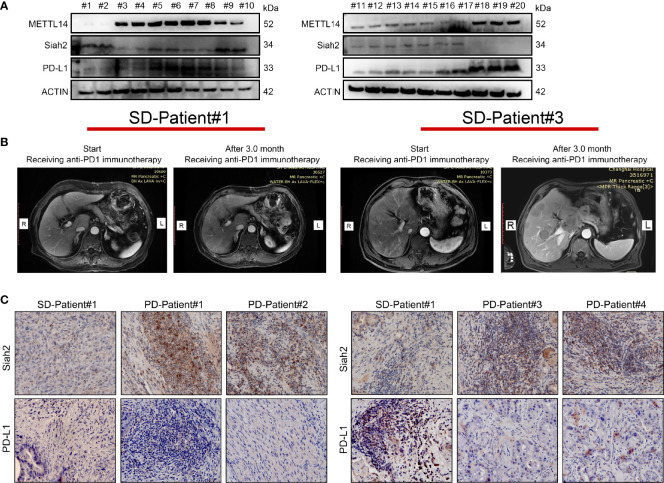
Correlation between METTL14, Siah2, and PD-L1 in clinical CCA specimens. **(A, B)**, Western blot analysis detected METTL14, Siah2, and PD-L1 protein levels in clinical CCA tumor samples. **(B)**, CT imaging of SD#1-CCA Patient and PD#3-CCA Patient. **(C)**, Siah2 and PD-L1 expression in the tumor area of CCA tissues detected by IHC. Magnification X 200. PD, progressive disease; SD, stable disease.

We investigated the clinical correlation between METL14, Siah2, and PD-L1 protein expression. We confirmed a negative correlation between Siah2 and PD-L1 protein level, a negative correlation between METL14 and Siah2, and a positive correlation between METL14 and PD-L1 in 30 human CCA tumor tissues by Western blot (**Figure 8A**). Next, we investigated whether Siah2 expression was associated with anti-PD-L1/PD1 immunotherapy response. We collected six specimens from patients before receiving anti-PD1 immunotherapy, and evaluated the therapeutic responses three months later [2/6 Stable Disease (SD), 4/6 Progressive Disease (PD); **Figure 8B**]. IHC detection of Siah2 and PD-L1 indicated that SD specimens displayed low Siah2 expression and PD-L1–positive expression on CCA tumor cells, and PD specimens displayed high Siah2 expression and PD-L1-negative expression (**Figure 8C**). These data showed that high expression of Siah2 may be an indicator for anti-PD-L1/PD1 immunotherapy response.

## Discussion

The low responsiveness of anti-PD-1/PD-L1 immunotherapy highlights the requirement of complete understanding of the PD-L1 regulation mechanism (27, 28). In this study, we reported for the first time a novel m6A modification of MEETL14-Siah2-PD-L1 axis in CCA. We found that Siah2 was regulated by an m6A modification in the mRNA, and the main m6A enzyme controlling Siah2 mRNA levels was METTL14. The significant correlation between METTL14 and Siah2 was confirmed in clinical CCA specimens by Western blotting and IHC. We also demonstrated the increased m6A modification on the 3’UTR region of the METTL4-induced PD-L1 mRNA in CCA cells. It has been documented that m6A modification affects mRNA stability, splicing, nuclear export, and/or translation of target mRNAs, depending on the distinct proteins that recognize them [e.g., YTHDF1, YTHDF2, YTHDF3 (6)]. We have shown that decreasing Siah2 mRNA levels by METTL14 were mainly due to the accelerated degradation of Siah2 mRNA, depending on the m6A reader protein YTHDF2, thereby decreasing Siah2 protein level. However, we did not evaluate whether m6A modifications affect the translation efficiency of Siah2, and worth further discussion. To the best of our knowledge, this is the first study to report the involvement of m6A modification in Siah2 dysregulation in cancer.

The activity of PD-1/PD-L1 is complex, since it is modulated by multiple processes, including gene transcription, posttranscriptional modifications, posttranslational modifications (PTMs), and exosome transport (29). PTMs (e.g., glycosylation, phosphorylation, ubiquitination, palmitoylation, SUMOylation, and acetylation) have been demonstrated to play an essential role in the modulation of protein stabilization and protein-protein interactions of the PD-1/PD-L1 axis (30–32). Siah2 is known to play an important role in tumorigenesis and cancer progression (18). Numerous studies have determined that Siah2 promotes tumorigenesis in a variety of human malignancies (18). However, several studies revealed that Siah2 exhibited tumor suppressor function by promoting the proteasome-mediated degradation of several oncoproteins, suggesting that Siah2 could exert its biological function depending on different stages of tumor development (18). Moreover, Siah2 is subject to complex regulation, especially the phosphorylation of Siah2 by a variety of protein kinases to regulate its stability and activity (18). However, the role of Siah2 in regulating tumor immunoenvironment in CCA, and the underlying molecular mechanism of this potential Siah2-regulated PD-L1 protein level has not been yet elucidated. We showed that Siah2 physically interacts with PD-L1 and increases K63-linked ubiquitination of PD-L1 in CCA. This finding expands the cognitive scope of the regulation mechanism of PD-L1 protein levels. To our knowledge, this is the first report concerning the role of Siah2 regulating PD-L1 protein level by K63-linked ubiquitination in CCA, and our results suggest the clinical significance of Siah2-PD-L1 axis in CCA Immunotherapy.

In the present study, we demonstrated that tumor-intrinsic Siah2-PD-L1 axis inhibited T cell–mediated cytotoxicity. The lack of a murine CCA cell line restricted our study on tumor-immune microenvironment to optimize immunotherapy in CCA. Therefore, we co-cultured activated HPBMC with human ICC cells *in vitro* and *in vivo* to investigate if the inhibitory effect of Siah2 on non-specific T-cell immunity was PD-L1-dependent. For deeper understanding of Siah2 role in ICC tumor immune microenvironment, we used CD34^+^ humanized NCG mice models, which have the same composition of immune cell subsets, especially regarding myeloid cells, which are considered to be a better preclinical model (33, 34). By applying CD34+ humanized NCG mice model and PCCA cell model analysis, we further found that Siah2 enhanced *in vivo* antitumor T-cell immunity in a PD-L1–dependent manner, unveiling a complex role of Siah2 in the tumor-immune microenvironment.

In addition, although the use of PD-L1 IHC assays for predicting anti-PD-1/PD-L1 immunotherapy response was approved in some cancers [e.g., pembrolizumab in non-small cell lung cancer (35)], most cancers showed inconsistencies between PD-L1 immunohistochemistry readout and response (36). PD-L1 predictive role is confounded by technical issues and by discrepancies in scoring systems (36, 37). N-linked glycosylation of PD-L1 blocks its recognition by IHC but Lee et al. improved the PD-L1 detection method by removing glycosylation (38). Combination of PD-L1 detection with other biomarkers (e.g., tumor mutational burden, tumor-infiltrating lymphocytes, and cytotoxic gene signatures) is also promising (39). We suggest that the addition of Siah2 might have addictive predictive value than PD-L1 IHC alone.

**Figure 9:**
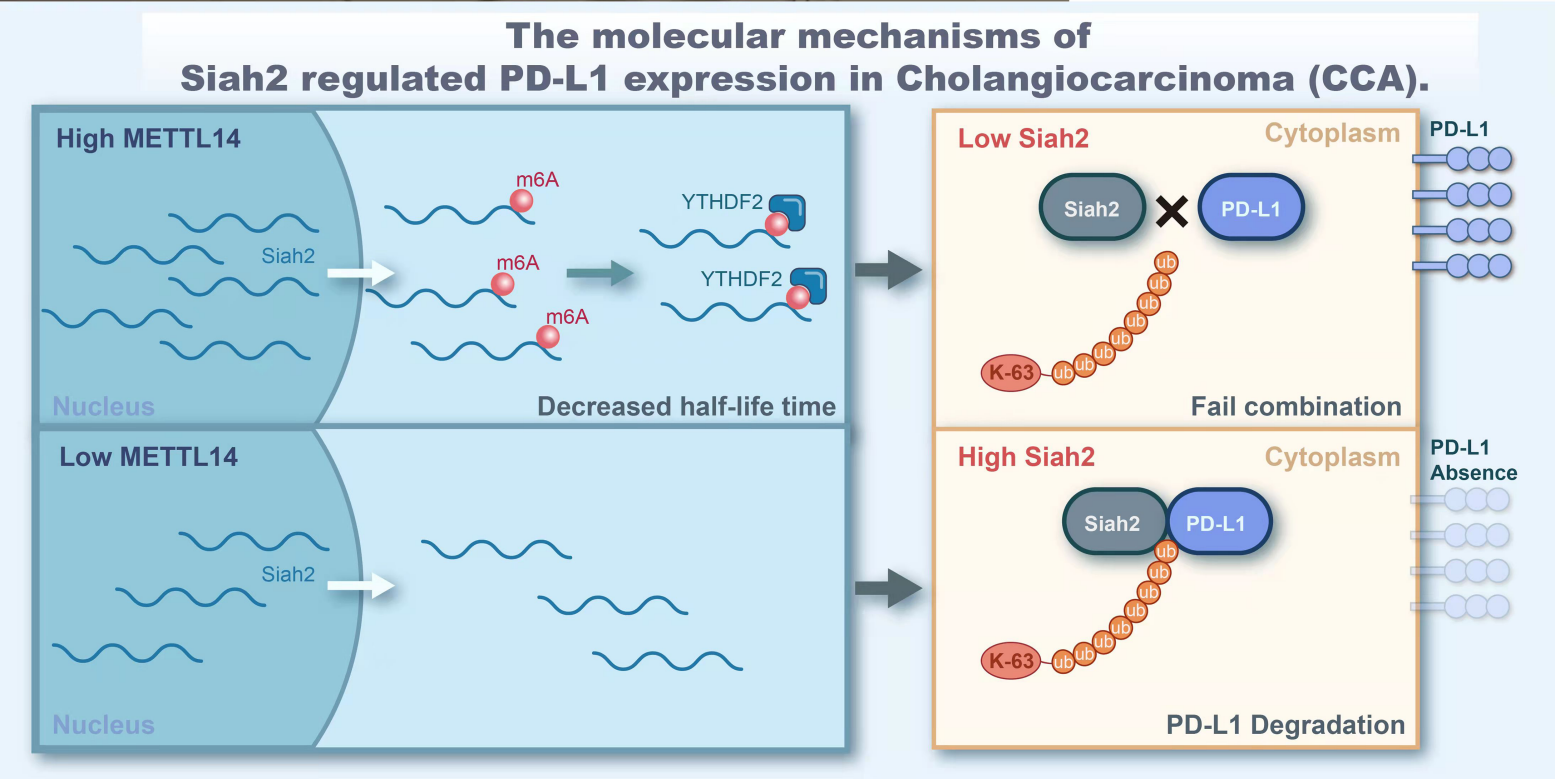
The molecular of Siah2 regulated PD-L1 expression in Cholangiocarcinoma (CCA).

In conclusion, our study demonstrated that Siah2 is a direct target of METTL14 and m6A modification. Mechanically, METTL14 enhances the m6A modification on 3’UTR region of Siah2 mRNA and mainly accelerates the degradation of Siah2 mRNA in an YTHDF2-dependent manner, thereby reducing Siah2 protein level. Siah2 physically interacts with PD-L1 and increases the K63-linked ubiquitination of PD-L1. Siah2 may enhance antitumor T-cell immunity through the Siah2-PD-L1 axis in CCA. Studies on specimens from patients before receiving anti-PD1 immunotherapy suggested that tumors with low Siah2 expression pattern might be more sensitive to anti-PD1 immunotherapy (**Figure 9**). Taken together, our study unveils the molecular mechanism of M6A-Siah2-PD-L1 axis in CAA, and extends the understanding of Siah2 in tumor-immune microenvironment and immunotherapy. However, this study still has some drawbacks and there is also knowledge worthy of further study, such as we lack a large-scale clinical cohort to verify the expression of mettl14, siah2, and PD-L1 and their correlation with clinical indicators and prognosis.

## Data Availability Statement

The raw data supporting the conclusions of this article will be made available by the authors, without undue reservation.

## Ethics Statement 

Tissue specimens were obtained from the Eastern Hepatobiliary Surgery Hospital (Shanghai, China) and the Changhai Hospital (Shanghai, China). The patients/participants provided their written informed consent to participate in this study. All animal experiments were conducted in conformity with conformity with NIH and the Second Affiliated Hospital of Nanchang University, Servicebio Animal Welfare guidelines and approved by Wuhan Servicebio Technology Co., Ltd., China. For experiments using human samples, written informed consent was obtained from patients. Written informed consent was obtained from the individual(s) for the publication of any potentially identifiable images or data included in this article.

## Author Contributions

HZ, Z-gW, and Y-pT performed data analysis and interpretation of results. All authors participated in planning and performing the experiments. LF, FZ, and BL conceived and planned the experiments. All authors contributed to the article and approved the submitted version.

## Funding

This work was supported by National Natural Science Foundation of China (No.82160578, No.81760438).

## Conflict of Interest

The authors declare that the research was conducted in the absence of any commercial or financial relationships that could be construed as a potential conflict of interest.

The editor CH declared a shared parent affiliation with the authors HZ, LO, GJ at the time of the review.

## Publisher’s Note

All claims expressed in this article are solely those of the authors and do not necessarily represent those of their affiliated organizations, or those of the publisher, the editors and the reviewers. Any product that may be evaluated in this article, or claim that may be made by its manufacturer, is not guaranteed or endorsed by the publisher.
