# Corrigendum: Decreased expression of programmed death ligand-L1 by seven in absentia homolog 2 in cholangiocarcinoma enhances T-cell–mediated antitumor activity

**DOI:** 10.3389/fimmu.2022.1093403

**Published:** 2022-12-08

**Authors:** Hao Zheng, Wen-juan Zheng, Zhen-guang Wang, Yuan-ping Tao, Zhi-ping Huang, Le Yang, Liu Ouyang, Zhi-qing Duan, Yi-nuo Zhang, Bo-ning Chen, Dai-min Xiang, Gang Jin, Lu Fang, Fan Zhou, Bo Liang

**Affiliations:** ^1^ Department of General Surgery, The Second Affiliated Hospital of Nanchang University, Nanchang, China; ^2^ Department of Reproductive Heredity Center, Changhai Hospital, Second Military Medical University, Shanghai, China; ^3^ Third Department of Hepatic Surgery, Eastern Hepatobiliary Surgery Hospital, Second Military Medical University, Shanghai, China; ^4^ Key Laboratory of Signaling Regulation and Targeting Therapy of Liver Cancer (SMMU), Ministry of Education, Shanghai, China; ^5^ Shanghai Key Laboratory of Hepatobiliary Tumor Biology (EHBH), Shanghai, China; ^6^ National Liver Tissue Bank, Eastern Hepatobiliary Surgery Hospital, Second Military Medical University, Shanghai, China; ^7^ Department of Hepatobiliary Surgery, General Hospital of Southern Theatre Command, Guangzhou, China; ^8^ Department of Hepatobiliary Pancreatic Surgery, Changhai Hospital of Second Military Medical University, Shanghai, China; ^9^ State Key Laboratory of Oncogenes and Related Genes, Shanghai Cancer Institute, Renji Hospital, Shanghai Jiao Tong University School of Medicine, Shanghai, China

**Keywords:** N6-methyladenosine, METTL14, Siah2, PD-L1, immunotherapy

In the published article, there was an error in **Figures 4**, **5** and **7** as published. The protein bands of SIAH2 in RBE-input groups in **Figure 4D** (leftmost panel) and the protein bands of SIAH2 in HUCCT1-IP-Flag groups in **Figure 4D** (right most panel) were duplicated from different exposure time. We have corrected the accurate protein bands of SIAH2 in HUCCT1-IP-Flag groups in **Figure 4D** (right most panel) by the raw data. The corrected **Figure 4** appears below.

**Figure 4:**
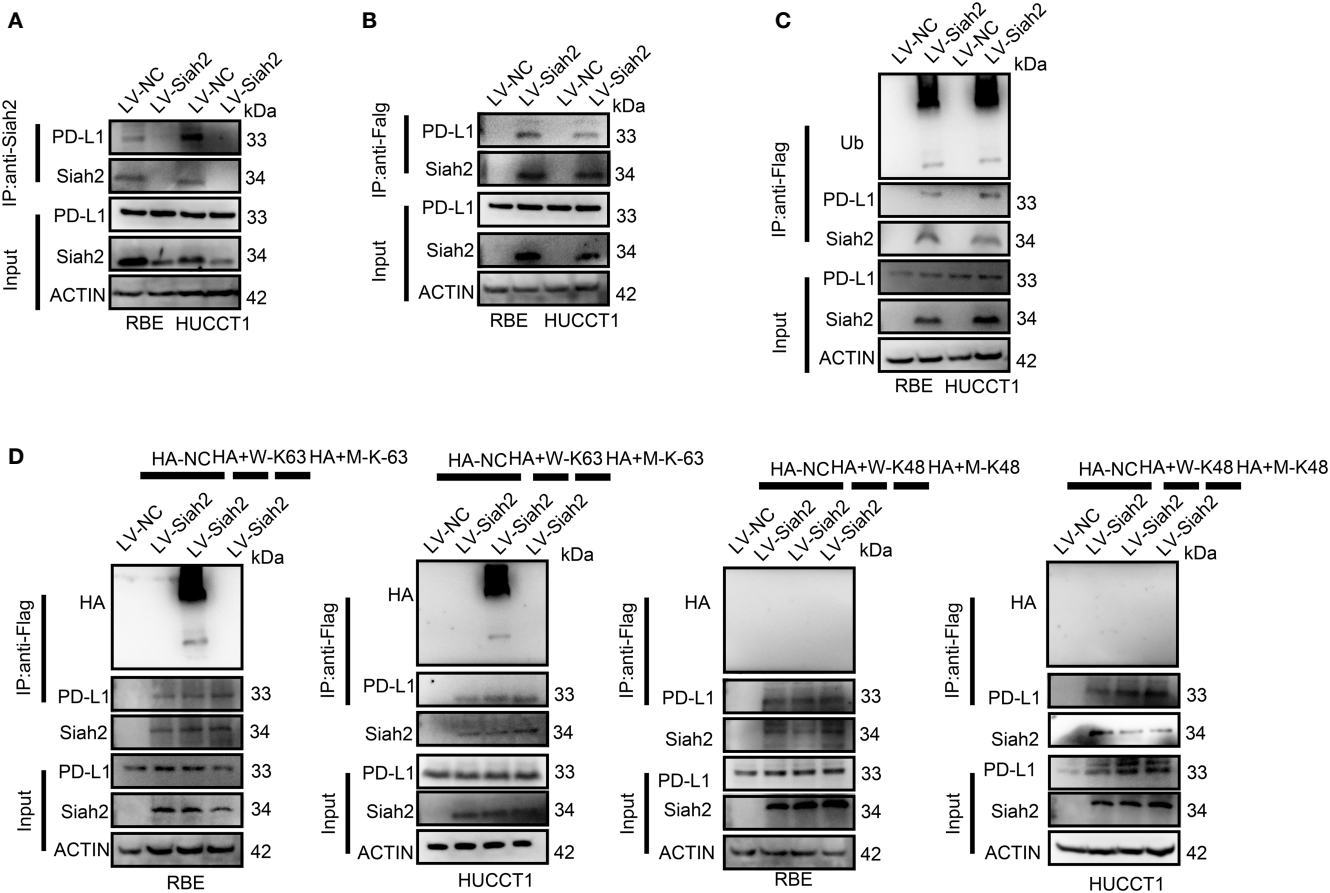
Siah2 physically interacts with PD-L1 and increases the K63-linked ubiquitination of PD-L1 in CCA. **(A)**, CCA cells were transfected with HA-Siah2 and Flag-PD-L1 for 36 h. Immunoprecipitation was performed with anti-Siah2 and analyzed by immunoblotting. **(B)**, CCA cells were transfected with HA-Siah2 and Flag-PD-L1 for 36 h. Immunoprecipitation was performed with anti-HA and analyzed by immunoblotting. **(C)**, CCA cells with LV-NC/LV-Siah2 were treated with MG132 for 8 h before harvesting, after which cells were harvested in lysis buffer. Supernatants were incubated with the appropriate antibodies overnight. Ubiquitin-modified proteins were analyzed with anti-Ub antibodies. **(D)**, CCA cells with LV-NC/LV-Siah2 were treated with MG132 for 8 h before harvesting, after which cells were harvested in lysis buffer. Supernatants were incubated with the appropriate antibodies overnight. Ubiquitin-modified proteins were analyzed with anti-lysine 48(K48)–linked polyubiquitination or K63-linked polyubiquitination.

**Figure 5:**
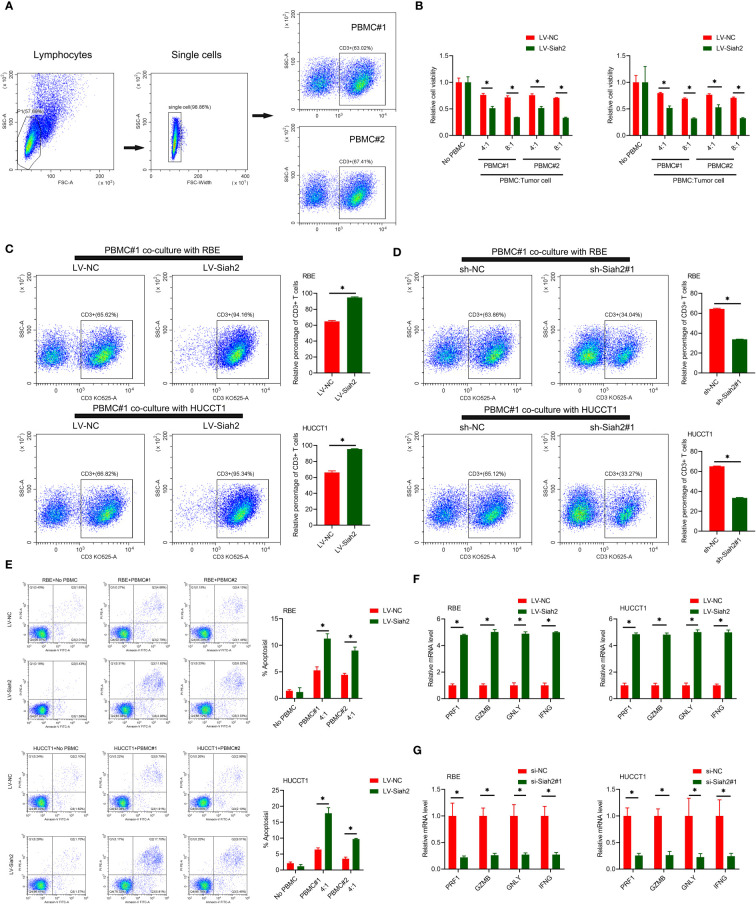
Siah2 enhanced in vitro antitumor T-cell activity. **(A)**, Gating strategy and T cell percentage of activated PBMC. **(B)**, CCK8 assay detected the killing of tumor cells by activated HPBMC. CCA cells were co-cultured in the presence or absence of HPBMC for 24 h. Data were normalized to their respective no HPBMC controls. **(C, D)**, Activated HPBMC (#1, #2) were co-cultured with LV-NC/LV-Siah2 **(C)** or sh-NC/sh-Siah2 **(D)** for three days at the ratio of HPBMC to tumor cell number of 4 to 1. The percentage of CD3 in HPBMC was determined by FCM. Representative plots of CD3+ T cells are shown. Data were normalized to the control group. **(E)**, Activated HPBMC (#1, #2) were co-cultured with CCA with LV-NC/LV-Siah2 cells for 24 h at the ratio of HPBMC to tumor cell number of 4 to 1. HPBMC were collected and stained with PE-Annexin V and subjected to FCM analysis. The percentage of apoptotic cells was analyzed. **(F, G)**, Quantitative RT-PCR was performed to detect PRF1 (perforin-1), GZMB (granzyme), GNLY (granulysin), and IFNG (IFN-g) in activated HPBMC co-cultured with CCA cells with LV-NC/LV-Siah2 **(G)** for the indicated time or with si-NC/si-Siah2 cells for 48 h. The ratio of HPBMC to tumor cell number was 4 to 1. Mean ± SEM of three independent experiments. *P < 0.05.

**Figure 7:**
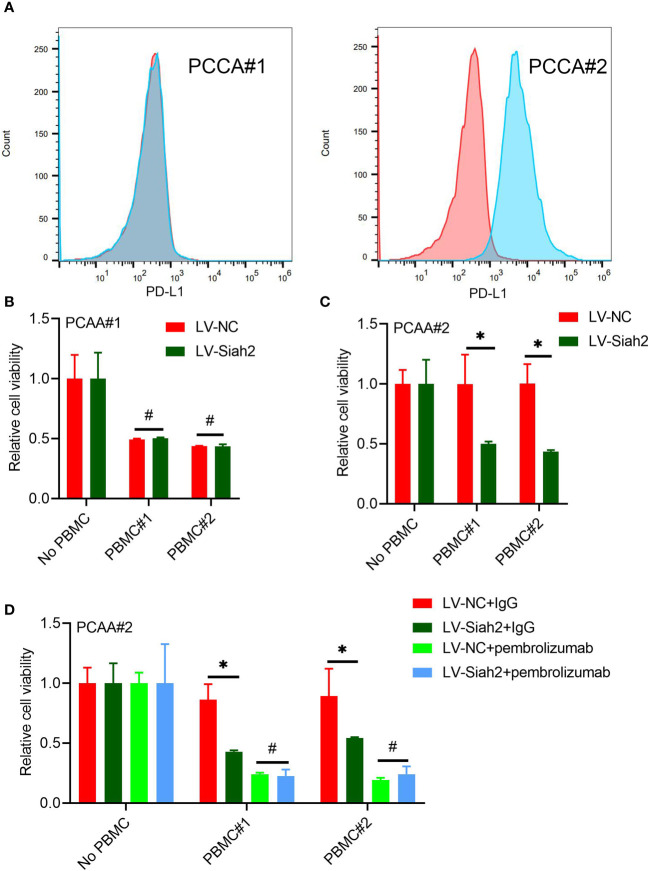
Siah2 enhanced antitumor T-cell immunity in a PD-L1–dependent manner. **(A)**, FCM detected the cell membranous PD-L1 level of PCCA#1 and #2. **(B, C)**, CCK8 assay detects the killing of PCAA#1 LV-NC/LV-Siah2 by activated HPBMC in PCCA#1 and PCCA#2 cells. The ratio of PBMC to tumor cell numberwas 4 to 1, n = 3. **(D)**, CCK8 assay detected the killing of tumor cells by activated HPBMC after Pembrolizumab or 10 μg/mL IgG treatment. PCAA#2-LV-NC/LVMETTL14 were co-cultured in the presence or absence of HPBMC for 24 h at the ratio of HPBMC to tumor cell number of 4 to 1, n = 3. Mean ± SEM of three independent experiments. *P < 0.05; #P > 0.05.

We uploaded the wrong version of incorrect representative flow cytometry graphs and quantitative data (**Figures 5A, C–E** and **Figure 7A**) owing to the raw data of flow cytometry analyzed disordered, and misplaced data was also due to our Flow experiment platform was changed. The corrected **Figures 5** and **7** were shown below.

In the published article, there was an error in the Funding statement. In the original text, this work was supported by the following institutes: (1) National Key Research and Development Program of China (2018YFC1004900, 2018YFC1005002); (2) National Natural Science Foundation of China (81672350, 81872225, 81871988, No.82160578, No.81760438). In fact, the funding of National Key Research and Development Program of China (2018YFC1004900, 2018YFC1005002) and Natural Science Foundation of China (81672350, 81872225, 81871988) should be deleted.

The correct Funding statement appears below.

This work was supported by National Natural Science Foundation of China (No.82160578, No.81760438).

In the published article, there was an error in the original article: Materials and Methods, CD34+ Humanized Mouse Models and Ethics statement.

A correction has been made to Materials and Methods, CD34+ Humanized Mouse Models, the description in this section corrected appears below:

## hPBMC+ Humanized mouse models

“hPBMC+ humanized NCG mice were purchased from the Model Animal Research Center of Nanjing University and were constructed as previously reported (25). Immune cell percentages were detected by flow cytometry 3 wk after hPBMC+ cell injection, hPBMC+ humanized NCG mice were randomly assigned into experiment groups. Indicated CCA cells with LV-NC/LV-SIAH2 of 5×10^6^ were injected into the right flank of hPBMC+ humanized NCG mice. Tumor volume was calculated by the following formula: volume = ab^2^/2 (a, the longer axis; b, the shorter axis). After 35 d of cell inoculation, hPBMC+ humanized NCG mice were euthanized and tumor-infiltrating leukocytes were isolated and subjected to CyTOF analysis. The animal study was conducted in conformity with NIH and the Second Affiliated Hospital of Nanchang University, Servicebio Animal Welfare guidelines and approved by Wuhan Servicebio Technology Co., Ltd., China”.

A correction has been made to Ethics Statement, Paragraph 4 and 5. This sentence previously stated: “All animal experiments were conducted in conformity with NIH guidelines and approved by the Ethics Committees of Naval Military Medical University.” The corrected sentence appears below: “All animal experiments were conducted in conformity with conformity with NIH and the Second Affiliated Hospital of Nanchang University, Servicebio Animal Welfare guidelines and approved by Wuhan Servicebio Technology Co., Ltd., China”.

In the published article, there was an error in Supplementary Table 1 and Table 3. In the original version accidently uploaded the error table Supplement Table 1, and SIAH2 primer sequences in Supplement Table 3 were written erroneously. The correct material statement appears below.

The authors apologize for these errors and state that this does not change the scientific conclusions of the article in any way. The original article has been updated.

## Conflict of interest

The authors declare that the research was conducted in the absence of any commercial or financial relationships that could be construed as a potential conflict of interest.

## Publisher’s note

All claims expressed in this article are solely those of the authors and do not necessarily represent those of their affiliated organizations, or those of the publisher, the editors and the reviewers. Any product that may be evaluated in this article, or claim that may be made by its manufacturer, is not guaranteed or endorsed by the publisher.

