# Supplementary material for: Decreased Expression of Programmed Death Ligand-L1 by Seven in Absentia Homolog 2 in Cholangiocarcinoma Enhances T-Cell–Mediated Antitumor Activity
Source: Front Immunol. 2022 Jan 27;13:845193. doi: 10.3389/fimmu.2022.845193 (PMC8828655; doi:10.3389/fimmu.2022.845193)
Supplement: Supplementary file 1 [file Table_1.pdf]

Decreased Expression of Programmed Death Ligand-L1 by Seven in Absentia Homolog 2 in Cholangiocarcinoma Enhances T-cell–mediated antitumor activity

Hao Zheng1,2,3,4,5#, Wen-juan Zheng1#, Zhen-guang Wang3,4，5#, Yuan-ping Tao6#, Zhi-ping Huang7#, Le Yang6, Liu Ouyang8, Zhi-qing Duan1, Yi-nuo Zhang1, Bo-ning Chen1, Dai-min Xiang9, Gang Jin8, Lu Fang1&, Fan Zhou1&, Bo Liang1&

1. Department of general surgery, The second affiliated hospital of Nanchang University, Nanchang，Jiangxi province, China.

2. Department of Reproductive Heredity Center, Changhai Hospital, Second Military Medical University, Shanghai, 200433, People's Republic of China.

3. Third Department of Hepatic Surgery, Eastern Hepatobiliary Surgery Hospital, Second Military Medical University, Shanghai, 200438, People's Republic of China.

4. Key Laboratory of Signaling Regulation and Targeting Therapy of Liver Cancer (SMMU), Ministry of Education, Shanghai, 200438, People's Republic of China.

5. Shanghai Key Laboratory of Hepatobiliary Tumor Biology (EHBH), Shanghai, 200438, People's Republic of China.

6. National Liver Tissue Bank, Eastern Hepatobiliary Surgery Hospital, Second Military Medical University, Shanghai, China.

7. Department of Hepatobiliary Surgery, General Hospital of Southern Theatre Command, 111 Liuhua Road, Guangzhou 510010, China.

8. Department of Hepatobiliary pancreatic surgery, Changhai Hospital of Second Military Medical University, Shanghai, 200433, China.

9. State Key Laboratory of Oncogenes and Related Genes, Shanghai Cancer Institute, Renji Hospital, Shanghai Jiao Tong University School of Medicine, Shanghai 200032, China.

Contribution equality

Correspondence to:

Lu Fang: Department of general surgery, The second affiliated hospital of Nanchang University, Nanchang，Jiangxi province, China. E-mail: fanglu@medmail.com.cn.

Fan Zhou: Department of general surgery, The second affiliated hospital of Nanchang

University, Nanchang， Jiangxi province, China. E-mail: nczhoufan@hotmail.com;.

Bo Liang: Department of general surgery, The second affiliated hospital of Nanchang University, Nanchang， Jiangxi province, China. E-mail: lb2087@163.com.

Supplement Materials and Methods

Patients and tissue specimens

The study protocol was approved by the clinical research ethics committee of the Eastern Hepatobiliary Surgery Hospital and Changhai Hospital. Written informed consent was obtained from all patients according to the policies of the committee. Any information that identifies patients was not included in this article. This study included 21 CCA tissues for N6-methyladenosine RNA immunoprecipitation, quantitative RT-PCR, and RT-PCR. The clinical characteristics of the HCC cohort are listed in **Table S4 (cohort-1)**. Additional 20 CCA tissues were evaluated by Western blotting and the clinical characteristics of the CCA cohort are listed in **Table S4 (cohort-2)**. Primary cells from two CCA patients' tumors were cultured as previously reported (1). All CCA specimens were obtained immediately after hepatectomy. Tissues were then fixed in 10 % buffered formalin and embedded in paraffin. Fresh specimens used in this study were snap-frozen from tissues prior to formalin fixation, transferred to liquid nitrogen, and stored at −80 °C, until use.

Cell culture and pharmacologic drug treatment

RBE is from National Laboratory Cell Resource Sharing Center (Beijing, China). HUCCT1 is from JCRB Cell Bank (Japanese Collection of Research Bioresources). Cells were cultured at 37 ºC in an atmosphere of 5% CO_2 in RPMI-1640 (GIBCO Laboratories, Grand Island, NY, USA) supplemented with 10% FBS (GIBCO) and 1% penicillin–streptomycin solution (GIBCO). The genetic identity of RBE and HUCCT1 were confirmed by short tandem repeat profiling. Cell lines are routinely tested for Mycoplasma contamination every three months.

Cycloheximide (CHX, protein synthesis inhibitor; MCE, catalog no. HY-12320), MG132 (proteasome inhibitor; Selleck, Shanghai, China, catalog no. S2619) and actinomycin D (an inhibitor of DNA transcription and replication; MCE, catalog no. HY-17559) were used at a final concentration of 10 mg/mL, 50 mmol/L, and 5 mg/mL, respectively. 3-Deazaadenosine (a global methylation inhibitor; MCE, catalog no. HY-W013332) was used at a final concentration of 0 to 50 mmol/L. Pembrolizumab (anti-

PD-L1 blockade antibody; Selleck, catalog no. A2004) or control IgG (BioXcell, West Lebanon, NH, USA; catalog no. BE0297) was used at 10 mg/mL for cell treatment.

N6-methyladenosine RNA immunoprecipitation quantitative RT-PCR of fragmented mRNA assay

Enrichment of m6A-modified mRNA was performed as previously reported (2,3). Immunoprecipitated m6A modified mRNA and"input"mRNA were subjected to quantitative RT-PCR with indicated primers. Fold enrichment was determined as the ratio of m6A modified mRNA to"input"mRNA.

Experimental animal models

For in vivo tumor growth assays, indicated treated CCA cells were subcutaneously injected at 2×10^6 cells per mouse into nude mice. Xenografted tumor growth was monitored as previously reported (4).

Co-Immunoprecipitation (IP)

For transient transfection and co-immunoprecipitation experiments, CCA cells (1×10^6) were transfected for 48 h. Transfected cells were lysed in 0.5 mL of the lysis buffer. For each immunoprecipitation, a 0.4 mL aliquot of the lysate was incubated with 4 μL of the indicated antibody or control IgG overnight at 4 °C, after which 20 μL of magnetic beads (Millipore, Billerica, MA, USA) were added for additional 2 h. Precipitates were then analyzed by standard Western blot procedures. The anti- primary antibody was listed in **Table S2**.

In vitro T-cell killing assay

In vitro T-cell killing assay was performed as previously reported (5). Pre-activated HPBMC were co-cultured with tumor cells. After incubation, the viability of tumor cells was measured by the Cell Counting Kit-8 (CCK8; Dojindo, Kumamoto, Japan) and apoptosis of tumor cells was detected by the Annexin VPE Apoptosis Detection Kit (Beyotime Biotechnology, Shanghai, China; catalog no. C1065S).

Supplement reference

1. Simon AG, Esser LK, Ellinger J, Branchi V, Tolkach Y, Müller S, et al. Targeting glycolysis with 2-deoxy-D-glucose sensitizes primary cell cultures of renal cell carcinoma to tyrosine kinase inhibitors. J Cancer Res Clin Oncol (2020) 146(9):2255-2265. doi: 10.1007/s00432-020-03278-8

2. Chen M, Wei L, Law CT, Tsang FH, Shen J, Cheng CL, et al. RNA N6-methyladenosine methyltransferase-like 3 promotes liver cancer progression through YTHDF2-dependent posttranscriptional silencing of SOCS2. Hepatology (2018) 67(6):2254-2270. doi: 10.1002/hep.29683

3. Lan T, Li H, Zhang D, Xu L, Liu H, Hao X, et al. KIAA1429 contributes to liver cancer progression through N6-methyladenosine-dependent post-transcriptional modification of GATA3. Mol Cancer (2019) 18(1):186. doi: 10.1186/s12943-019-1106-z

4. Ma YN, Hong YG, Yu GY, Jiang SY, Zhao BL, Guo A, et al. LncRNA LBX2-AS1 promotes colorectal cancer progression and 5-fluorouracil resistance. Cancer Cell Int (2021) 21(1):501. doi: 10.1186/s12935-021-02209-y

5. Qiu X, Yang S, Wang S, Wu J, Zheng B, Wang K, et al. M(6)A Demethylase ALKBH5 Regulates PD-L1 Expression and Tumor Immunoenvironment in Intrahepatic Cholangiocarcinoma. Cancer Res (2021) 81(18):4778-4793. doi: 10.1158/0008-5472.CAN-21-0468

Supplement Table 1. shRNA-lentivirus seed sequences.

Name	Seed sequence（5‘-3’）
sh-NC	GAAGGTCGGAGTCAACGGATTC
sh-METTL14#1	GAAGATATTTGTTGGATTAAAC
sh-METTL14#2	ACATTGACTTAATTATCACAGC
sh-YTHDF2#1	GCAAAACAGCAACCTAAACTGC
sh-YTHDF2#2	CTACTCTGAGGACGATATTCAC
sh-SIAH2#1	GTCATGTTTTGGCCATCACTTC
sh-SIAH2#2	AACCTTGGAATCAATGTTACTC

Supplement Table 2. Primary antibodies.

Antigens	Manufacturer	Application
FTO	Abcam	For WB 1:1000
METTL3	Abcam	For WB 1:1000
METTL14	Abcam	For WB 1:1000
ALKBH5	Abcam	For WB 1:1000
ACTIN	Abcam	For WB 1:10000
Siah2	Abcam	For WB 1:1000
YTHDF2	Abcam	For WB 1:1000
PD-L1	Abcam	For WB 1:1000
Anti-Ubiquitin (Linkage-specific K48)	Abcam	For WB 1:10000
Flag	Abcam	For WB 1:1000
Anti-Ubiquitin (Linkage-specific K63)	Abcam	For WB 1:1000
Anti-Ubiquitin (Linkage-specific K48)	Abcam	For WB 1:1000
Siah2	Abcam	For IHC 1:50
PD-L1	Abcam	For IHC 1:50

Supplement Table 3. Quantitative RT-PCR primers.

Primer name	Property	Sequence
Siah2	Forward (5′-3′)	5′-TAACCAATGCCGCCAGAAGT-3′
	Reverse (5′-3′)	5′-CCCGTGGTGGCATACTTACA-3′
PD-L1	Forward (5′-3′)	5′-TGGCATTTGCTGAACGCATTT-3′
	Reverse (5′-3′)	5′-TGCAGCCAGGTCTAATTGTTTT-3′
ACTIN	Forward (5′-3′)	5′-TGACGGGGTCACCCACACTG-3′
	Reverse (5′-3′)	5′-AAGCTGTAGCCGCGCTCGGT-3′
PRF1	Forward (5′-3′)	5′-GGCTGGACGTGACTCCTAAG-3′
	Reverse (5′-3′)	5′-CTGGGTGGAGGCGTTGAAG-3′
GZMB	Forward (5′-3′)	5′-CCCTGGGAAAACACTCACACA-3′
	Reverse (5′-3′)	5′-GCACAACTCAATGGTACTGTCG-3′
GNLY	Forward (5′-3′)	5′-CAGGCTCCCTGCCCATAAAA-3′
	Reverse (5′-3′)	5′-CTCAAGGCCTGGGTTGCC-3′
IFN	Forward (5′-3′)	5′-TCGGTAACTGACTTGAATGTCCA-3′
	Reverse (5′-3′)	5′-TCGCTTCCCTGTTTTAGCTGC-3′
YTHDF2	Forward (5′-3′)	5′-AGCCCCACTTCCTACCAGATG-3′
	Reverse (5′-3′)	5′-TGAGAACTGTTATTTCCCCATGC-3′

Supplement Table 4. Clinicopathological characteristics of CCA patients in the study cohort.

**Clinicopathological Feature**		**cohort-1**	**cohort-2**
		**21**	**20**
**Gender**	Male	15	13
	Female	6	7
**Age, Year**	≥60	17	15
	<60	4	5
**Tumor size，cm**	≥5	6	7
	<5	15	13
**Tumor number**	Multiple	4	5
	single	17	15
**Lymph node metastasis**	Absent	15	14
	Present	6	6
**Vascular invasion**	Absent	18	17
	Present	3	3
**CEA, μg/L**	≥ 5	10	11
	< 5	10	9
**CA199, U/ml**	≥ 40	16	12
	< 40	5	8
**CA125, U/ml**	≥ 35	9	10
	< 35	12	10
**AFP，μg/L**	≥ 25	15	15
	< 25	6	5
